# Dichotomous associations of liver pathology with hepatocellular carcinoma morphology in Middle Africa: the situation in Cameroon

**DOI:** 10.1186/s13104-018-3560-x

**Published:** 2018-07-09

**Authors:** Marie Atsama Amougou, Paul Jean Adrien Atangana, Alice Ghislaine Ndoumba Afouba, Paul Fewou Moundipa, Pascal Pineau, Richard Njouom

**Affiliations:** 1 Virology Unit, Centre Pasteur of Cameroon, BP 1274 Yaoundé, Cameroon; 2 Laboratory of Pharmacology and Toxicology, University of Yaounde I, BP 815 Yaounde, Cameroon; 3 Unité « Organisation nucléaire et Oncogenèse », INSERM U993, Institut Pasteur, Paris, 75015 Paris, France

**Keywords:** Hepatocellular carcinoma, Liver biopsy, Hepatoblastoma, Steatosis, Trabecular, Cameroon

## Abstract

**Objective:**

This study evaluates the occurrence of the various morphological subtypes of hepatocellular carcinoma (HCC) and their connections with some risk factors in Cameroonian patients. The database of the 360 liver biopsies received and associated medical records were reviewed for histological and demographic analysis. Archival formalin-fixed and paraffin embedded liver biopsy specimens or slide were re-evaluated in malignancies patients. HCC classification was determined according to the World Health Organization criteria.

**Results:**

Malignancies were confirmed in 24.7% (89/360) of liver biopsies. Primary liver tumors consisted in 80 cases of HCC and one case of hepatoblastoma. The distribution of the morphological variants of HCC was trabecular pattern (n = 45/80, 56.25%), acinar/pseudoglandular (32.5%) or scirrhous (11.2%). Remarkably, liver steatosis was present in 60.0% (48/80) of patients with HCC, most of them infected with hepatitis C virus (75.8%). Well-differentiated trabecular tumors were significantly associated with important fibrotic and necro-inflammatory activities in livers (P = 0.008) whereas acinar pattern was more frequent on fatty livers (P = 0.02). Our finding indicates that in Middle Africa the morphology of HCC subtypes correlates with changes affecting non-tumor liver tissue. Trabecular subtype is installed by strong liver injury whereas acinar pattern is more often associated with lipid metabolism defects.

**Electronic supplementary material:**

The online version of this article (10.1186/s13104-018-3560-x) contains supplementary material, which is available to authorized users.

## Introduction

Primary liver cancer (PLC) is associated with a high mortality rate and it is the second cause of cancer-related death worldwide [1]. Sub Saharan Africa (SSA) represents a major region both in terms of incidence and mortality from HCC, the principal form of PLC [2, 3]. In Cameroon, little is known about the cancer burden in general. According to the GLOBOCAN 2012, however, PLC is the second type of cancers in term of incidence and the first cause of cancer-related mortality in the country [4].

Globally, the two main histological types of PLC are hepatocellular carcinoma (HCC) and cholangiocarcinoma (CCA) [5]. Of these two types, HCC is known as the leading form of liver cancer worldwide [6]. In addition, HCC is a heterogeneous disease with four majors morphological subtypes such as trabecular, pseudoglandular/acinar or solid patterns [7]. It is now clear that hepatitis B virus (HBV) and hepatitis C virus are the main etiological viral factor for HCC development worldwide [8, 9] and both viruses are highly endemic in middle Africa [10–12].

No recent study describing the distribution of PLC histotypes and morphologies is available in the country. Furthermore, despite the importance of HCC in the region, recent pathological reports about this tumor are scarce in SSA as a whole. Such descriptions are all the more important that the global epidemiology of HCC is currently changing with the growing importance taken by dysmetabolic conditions such as liver steatosis and non-alcoholic steatohepatitis among major risk factors [13–16]. The importance of these conditions is poorly known in SSA despite the ongoing nutritional transition that concern segments of the African populations and the growing prevalence of non-communicable diseases such diabetes and obesity. The aim of the present study was, thus, to fill this gap using liver biopsies diagnosed over a 10-year period in Yaounde.

## Main text

### Materials and methods

#### Data collection and inclusion criteria

This retrospective study was conducted in the Pathology Unit of Centre Pasteur of Cameroon recognized as the national reference Laboratory of the Country. This study was the continuity of another project on primary liver Cancers approved by the Cameroonian Ethics Commitee and the Ministry of Health. All the biopsies received from January 2004 to January 2013 were reviewed and revealed a total of 2068 biopsies. Only the 360 patients identified with liver biopsy were include in the present study. Histological and demographic data regarding age at diagnosis and sex information were obtained from medical record and the database of the Unit. The study did not include information regarding clinical features concerning patients affected with the tumors because these data were not available in many cases. All histological evaluation was performed by the single experienced pathologist and the histological residents of the Unit.

#### Histological evaluation

For histological diagnosis, slides were retrieved from the archives of the department and red first by the histological resident and finally by the single experienced pathologist of the Unit. Microscopic evaluation of the multiples selected sections was accomplish from sections of paraffin embedded tissue mounted on glass and stained with Hematin-Eosin and Masson Trichrome. The size and the quality (at least 3 portal spaces) of the biopsy specimens were noted. The liver fibrosis and necro-inflammatory activity were assessed using the METAVIR scoring system [17]. Fibrosis was therefore scored on a scale from 0 to 4 (F0 = no fibrosis, F1 = portal fibrosis without septa, F2 = portal fibrosis and few septa, F3 = numerous septa without cirrhosis and F4 = cirrhosis) and activity on a scale from 0 to 3 (A0 = none, A1 = mild, A2 = moderate and A3 = severe). HCC cases were further group into morphological sub-types as described according to WHO classification of tumors [18]. One representative paraffin-embedded section from each case was selected to determine the grade of steatosis represented by the percentage of hepatocytes containing fat droplets [19].

#### Statistical analysis

Data were represented as mean ± standard deviation (SD). Descriptive analysis was performed to characterize the demographic variables of the patients. An independent *t* test or nonparametric test was used to determine the difference between groups. Frequencies and proportions were used for categorical variables. The differences were determined by Chi square test or a Fisher exact test. The difference was considered statistically significant for P < 0.05. All tests were two-sided. Analyses were performed using SPSS 16.0 and Prism 6.0 statistical softwares.

### Results

During a 10-year period (January 2004 to January 2013) a total of 2068 of different types of biopsies were received and a total of 360 (17.4%) liver biopsies were included and examined. Among these, 25.8% (93/360) have been practiced on liver masses suspected to be hepatic malignancies. Primary liver cancer (PLC) was found in 87.0% (81/93) of them. For the remaining 12 specimens, metastatic tumors (5 adenocarcinomas and 1 lymphoma) represented 6.4% (6/93) of cases, mesenchymal tumors were found in two patients (angiosarcoma and neurofibroma, 2.3%) whereas kysts or inflammatory pseudo-tumors composed the last 4 cases (4.3%). The different types of malignancies found in this study are presented in Table 1.

**Table 1:** Summarization of demographic and clinico-pathological features of patients with HCC and those with hepatitis

Clinico-biological features	HCC n = 80	Hepatitis n = 247	P values
Age (mean ± SD)	38.1 ± 15.2	35.6 ± 12.5	0.21 (ns)
Sex ratio M:F	1.9 (52/28)	2.04 (166/81)	ns
Risk factors (%)			
HBV	52.5%	5.8%	0.008
HCV	36.5%	38.0%	ns
Unknown risk	11.2%	56.2%	3.07E−5
Steatosis (%)			
Total	60.0	5.9	
HBV	23.5	1.2	
HCV	75.5	4.7	
Unknown risk	1.01	00	na
Morphology			
Microtrabecular	42.6	–	
Macrotrabecular	13.7	–	na
Acinar-pseudoglandular	32.5	–	
Scirrhous	11.2	–	
Fibrosis	n = 73	n = 210	
F0	5.4	9.5	
F1	9.5	52.8	
F2	38.3	28.5	0.0321
F3	24.6	5.7	8.8 E−14
F4	20.5	3.3	3.9 E−6
Necro-inflammation	n = 74	n = 237	
A0	2.7	3.3	
A1	33.8	37.1	ns
A2	41.9	45.9	ns
A3	20.2	13.5	0.0334
Cellular variant			
Clear cells	12.5	24.9	0.0279
Ballooning degeneration	3.7	25.9	4.3 E−6

*HCC* hepatocellular carcinoma, *HBV* hepatitis B virus, *HCV* hepatitis C virus, *F0* no fibrosis, *F1* portal fibrosis without septa, *F2* portal fibrosis and few septa, *F3* numerous septa without cirrhosis, *F4* cirrhosis, *A0* none, *A1* mild, *A2* moderate, *et A3* severe, *M* male, *F* female, *ns* not significant, *na* not available

Two types of PLC were found, HCC representing 98.8% (n = 80/81) and hepatoblastoma (one case, 1.2%). The mean age of patients diagnosed with HCC was 38.1 ± 15.2 years (median = 33) with the peak of HCC occurrence between 20 and 39 years age (n = 49/80, 61.3%) and males were predominant among HCC patients 65% (n = 52/80). Demographic characteristics of patients diagnosed with HCC are presented in Table 1.

As resume in Table 1, a total of 42 HCC patients (52.50%) were found with HBV infection whereas 36.2% (29/80) were found with HCV infection. The etiology was unknown for 9 (11.5%) HCC patients and the only 2 years-old girl with hepatoblastoma.

The distribution of the morphological subtypes of HCC are presented in Table 1 and Additional file 1. In the present study, trabecular patterns were prevalently found (n = 45/80; 56.25%) whereas scirrhous subtypes were found in (n = 9/80; 11.25%) and acinar/pseudoglandular in (n = 26/80; 32.5%).

A minor subset of tumor cases 20.8% (15/72) occurred on cirrhotic livers while another 25% (n = 18/72) displayed severe fibrosis stage at F3. Necro-inflammatory activity was scored as moderate-to-severe (A2–A3) in 63.0% of HCC cases (Table 1). In addition, 60.2% of patients with HCC presented liver steatosis with four of them displaying more than 20% of hepatocyte involvement. Finally, two cytological features differed significantly between HCC and hepatitis. Clear cell variants and ballooning degeneration were less prevalent in tumors than in non-tumor samples (Table 1).

**Fig. 1:**
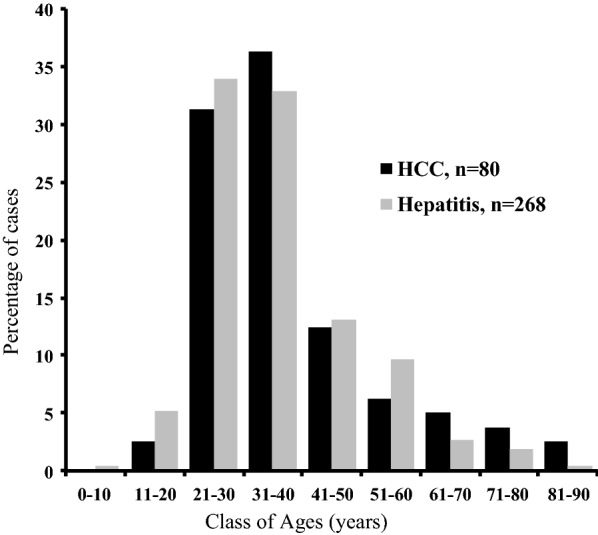
The distribution of age between patients with HCC and patients biopsied for hepatitis HCC: hepatocellular carcinoma

We subsequently tried to find clinico-biological correlations that might explain the different forms taken by tumors found in Cameroonian patients. As presented in Fig. 1, infectious risk factors were important determinant for the age of patients with HCC with HBV-infected being the youngest (33.1 ± 10.5), the nonBnonC the oldest (51.7 ± 13.2) and HCV-infected subjects occupying an intermediate position (41.5 ± 1.1).

As expected, HCV infection was associated with two well-known hallmarks of its histological impact on the liver. Severe Fibrosis scores (F3-F4) were significantly more prevalent in case of infection with this agent than without it (65.5 vs 30.0%, OR = 4.4, 95% CI 1.6–12.3, P = 0.0011). Similarly, liver steatosis was present in 75.8% of the livers from HCV-infected patients whereas it was observed in only 48.9% of others (OR = 3.2, 95% CI 1.1–9.0, P = 0.031). The reverse was at least true concerning fibrosis stages for HBV, which presence was less frequent in F3-F4 than in F0-F2 livers (36.3 vs 71.9%, OR = 0.22, 95% CI 0.08–0.60, P = 0.0041).

**Fig. 2:**
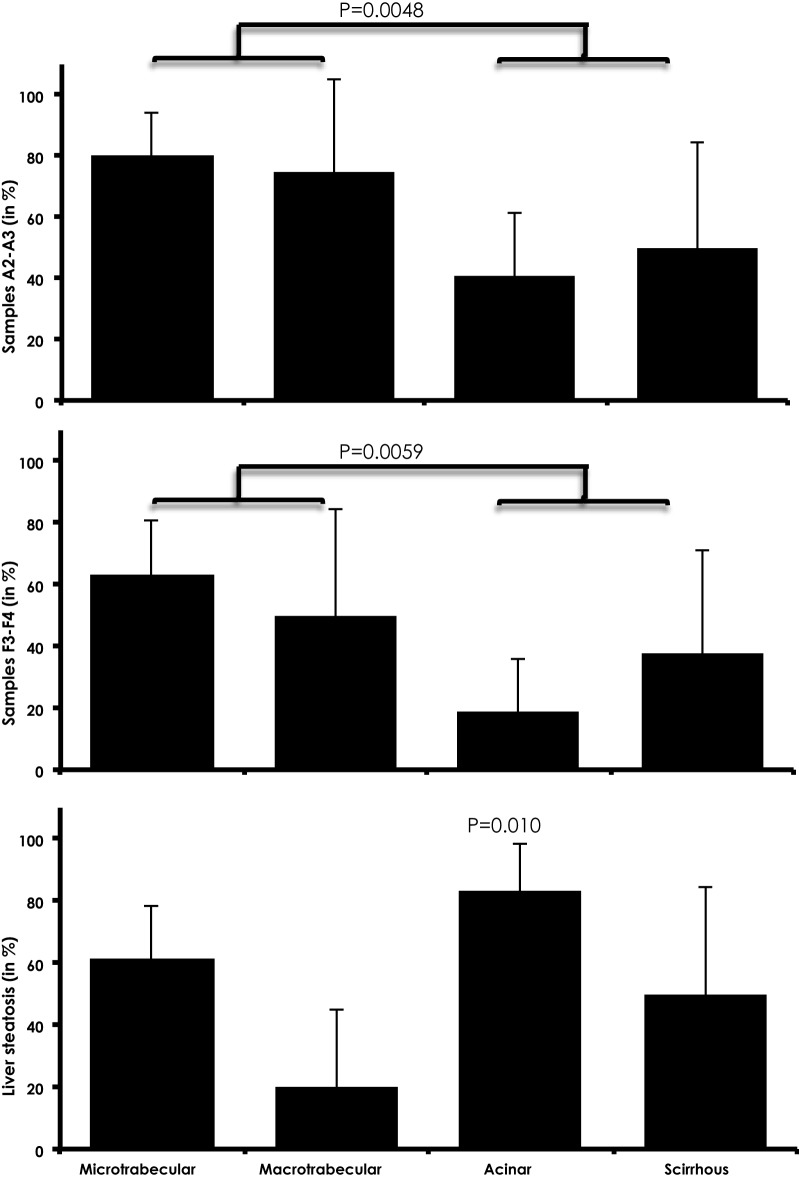
The distribution of the morphological variants of Hepatocellular carcinoma according to histological liver feature (steatosis, fibrosis and liver activity). *F0* no fibrosis, *F1* portal fibrosis without septa, *F2* portal fibrosis and few septa, *F3* numerous septa without cirrhosis, *F4* cirrhosis

Analyses of histological features were informative as well. Interestingly, characters of the underlying pathology affecting non-tumor livers were apparently influencing tumor morphology. Of course, necro-inflammatory activity and fibrosis were positively correlated. Indeed, tissues scored as A2–A3 were more often the siege of a F3–F4 fibrosis than A0–A1 specimens (74.4 vs 0.0%, OR = 156.2, P < 0.0001). More interestingly, we observed that trabecular HCC specimens were more often developing from non-tumor livers with intense A2–A3 necro-inflammatory (78.9 vs 43.3%, P = 0.0048) or with severe F3–F4 fibrosis (60.5 vs 24.1%, P = 0.0059; Fig. 2). By contrast, when the non-tumor liver was the siege of steatosis, tumor morphology was more frequently acinar/pseudoglandular instead (83.3 vs 51.0%, OR = 4.8, 95% CI: 1.4–16.1, P = 0.0101, Fig. 2). These two associations, high activity-fibrosis/trabecular pattern and steatosis/acinar pattern tended to be mutually exclusive as the presence of high activity and pervasive fibrosis were negatively correlated with acinar/pseudoglandular pattern (P < 0.008 for both features). In parallel, steatosis was strongly anti-correlating with the macrotrabecular morphology (4.4 vs 28.5%, P = 0.018).

### Discussion

This study identified the different morpho-types of HCC diagnosed in Cameroon through a retrospective analysis in the Pathology Unit of the Centre Pasteur of Cameroon from January 2004 to January 2013. It is the first review on the distribution of the different types of PLC in Cameroon.

HCC represents an overwhelming majority of the PLC recorded (98.8%), as only one hepatoblastoma case was reported (1.2%) in the corresponding period. Others types of PLC such as cholangiocarcinoma (CCA) were not diagnosed in the present study thus confirming that this tumor type did not emerged recently in SSA. This observation suggests that the pattern of tumor development remains stable since decades in the region. This is particularly interesting regarding the role of HCV, often suspected to cause the development of a sizeable subset of intra-hepatic CCA cases elsewhere [20]. HCV-associated biliary cells transformation is apparently still infrequent in Sub Saharan Africa. Our results indicate that HCC biopsies occur predominantly among 20 to 39-years-old adults in Cameroon. This age distribution is much younger than what is commonly reported in SSA for HCC as a whole. This phenomenon is presumably due to the feasibility of liver biopsy in younger patients. Indeed, liver tissues and functions including clotting factors production are often deteriorating with age making biopsies more at risk of complications and thus contraindicated [21–23]. A similar distortion, attributable to similar reasons, was also conspicuous regarding the prevalence of liver cirrhosis (20%) in the present series.

The present work provides data on the frequency of the histological sub-types of HCC. Trabecular variants (micro- or macro-) were the most commonly observed patterns in the present series (56.3% when take together). These results are consistent with studies conducted in South-Eastern Nigeria (49.3%) [24], in Zambia (52.9%) [25], in Tanzania (47.9%) [26] and Zaire (31.4%) [23]. Our results contrast however with a study conducted in Bangladesh where pseudo glandular subtype of HCC was the most prevalent [27, 28]. The prevalence of acinar/pseudoglandular morphotype in our series (32%) is grossly similar to that recently observed in Tanzania (25%) or Zambia (23%) but exceeds largely the proportion observed in North-Central Nigeria (13%). The causes such major differences in pattern distribution are unknown.

In the current report, 58.8% of HCC patients were presenting a certain degree of liver steatosis with more than 70% of these cases infected with HCV. The concomitance of a fatty liver and HCC used to be rather rare both in East or West Africa in reports published several decades ago [29–31]. Publications reporting sizeable proportions of fatty metamorphosis in liver tissues from Africans tend to appear recently though [32–34]. The presence of fatty degeneration to such extent in a series of HCC from the XXIst century might therefore represent a major evolution of liver pathophysiology and HCC epidemiology in SSA [35, 36].

### Conclusion

Our study reports for the first time the incidence of the different types of morphologies taken by HCC in Cameroon. Our results showed that the trabecular subtype is the predominant presentation but acinar/pseudo-glandular pattern is another important presentation of HCC in the country. Our study also reports a remarkable binary presentation of HCC and corresponding liver tissues with trabecular tumors arising mostly from fibrotic livers whereas acinar specimens were primarily observed in fatty livers. Further investigations needs to be conducted to identify micro-environmental mechanisms that promote these preferential associations.

## Limitations

The first one is that biological data, clinical or pathological stages of the tumors and detailed risk factors associated to the different types of HCC such as HBV DNA and HCV RNA loads were not available. Finally our survey is characterized by shortcomings inherent to cross-sectional observational studies and as such cannot compete with true case–control studies. However, it has the merit to provide an up-to-date landscape on the distribution and epidemiology associated with the different types of HCC in Cameroon. Further prospective studies are needed to confirm our results.

## Additional file

**Additional file 1.** Microphotographs of the **A**: (H&E × 10) liver parenchyma within the limits of normal but showing a slight dilation of sinusoids; **B**: (H&E × 10) and **C**: (H&E × 20) chronic hepatitis with moderate activity characterized by ballonnisation, cellular clarification of moderate intensity and the presence of macrovascular steatosis less than 20%; the morphological variants of HCC. **D:** (H&E × 4) and **E**: (H&E × 20) Moderately differentiated HCC with trabecular/acinar pattern; **F:** Moderately differentiated HCC with moderately to severe steatosis pattern (H&E × 40). *H&E* Hematin-Eosin.

## Abbreviations

HCC: hepatocellular carcinoma
HBV: hepatitis B virus
HCV: hepatitis C virus
SSA: Sub Saharan Africa
PLC: primary liver cancer
F0: no fibrosis
F1: portal fibrosis without septa
F2: portal fibrosis and few septa
F3: numerous septa without cirrhosis
F4: cirrhosis
A0: none
A1: mild
A2: moderate
et A3: severe
ns: not significant
na: not available
