# Supplementary figures and images for: Dichotomous associations of liver pathology with hepatocellular carcinoma morphology in Middle Africa: the situation in Cameroon
Source: BMC Res Notes. 2018 Jul 9;11:451. doi: 10.1186/s13104-018-3560-x (PMC6038295; doi:10.1186/s13104-018-3560-x)

## Slide 1
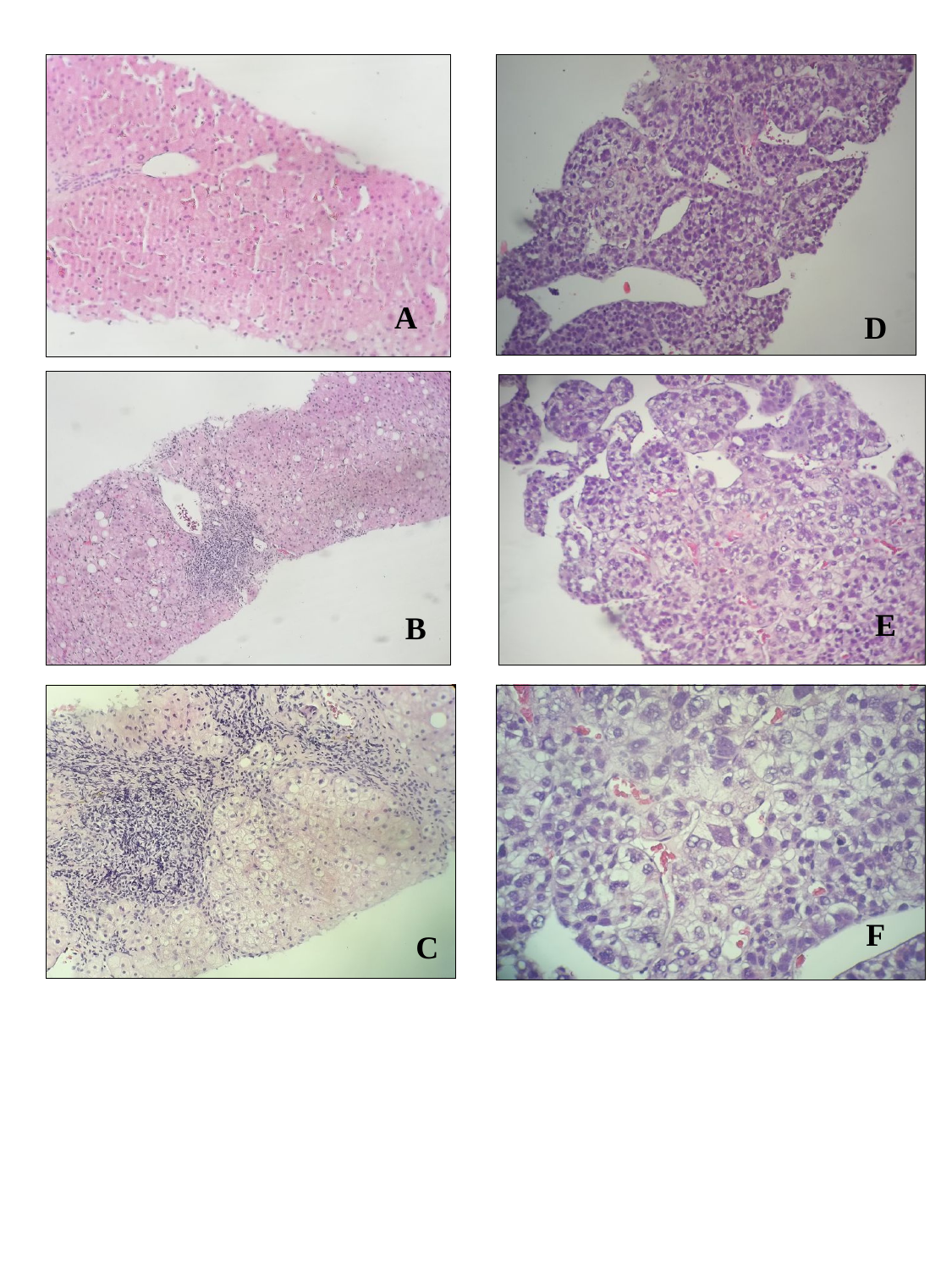

A
D
E
B
F
C

Supplement: Supplementary file 1 — Additional file 1. Microphotographs of the A: (H&E × 10) liver parenchyma within the limits of normal but showing a slight dilation of sinusoids; B: (H&E × 10) and C: (H&E × 20) chronic hepatitis with moderate activity characterized by ballonnisation, cellular clarification of moderate intensity and the presence of macrovascular steatosis less than 20%; the morphological variants of HCC. D: (H&E × 4) and E: (H&E × 20) Moderately differentiated HCC with trabecular/acinar pattern; F: Moderately differentiated HCC with moderately to severe steatosis pattern (H&E × 40). H&E Hematin-Eosin. [file 13104_2018_3560_MOESM1_ESM.pptx]
